# The Protective Effects of Microbe Derived Antioxidants on Digestive Tissue Morphology, Functions, and Intestinal Microbiota Diversity of *Eriocheir sinensis* Exposed to Glyphosate

**DOI:** 10.1155/anu/2620217

**Published:** 2024-12-10

**Authors:** Yameng Song, Mengyao Wu, Yongxu Cheng, Chao Niu, Xiaowen Yu, Yangyang Pang, Xiaozhen Yang

**Affiliations:** National Demonstration Center for Experimental Fisheries Science Education; Key Laboratory of Freshwater Aquatic Genetic Resources, Ministry of Agriculture; Engineering Research Center of Aquaculture, Shanghai Ocean University, No. 999 Huchenghuan Road, Shanghai 201306, China

**Keywords:** antioxidant, digestive enzymes, *Eriocheir sinensis*, glyphosate, histomorphometery, intestinal microbiota

## Abstract

**Introduction:** The use of glyphosate (Gly) has caused unnecessary economic losses to the aquaculture industry, but research on the effect of Gly on *Eriocheir sinensis* is very limited. The aim of this study is to reduce the negative effects of Gly, reduce yield loss, and improve economic benefits through nutritional feed control technology.

**Methods:** The experiment involved 80 crabs randomly divided into four groups: control group, Gly group (48.945 mg/L), microbe-derived antioxidant (MA) group, and Gly and MA treatment group. The study lasted for 7 days. In this study, the effects of Gly on the digestive function of *E. sinensis* were investigated using histology and spectrophotometer, and the gut microorganisms of *E. sinensis* were analyzed using high-throughput sequencing technology.

**Results:** The study found that exposure to Gly resulted in separation of the folds of the midgut mucosa of *Eriocheir sinensis* from the basement membrane, a decrease in the fold area of the hindgut mucosa, and an increase in the number of B cells in hepatic tubules. Additionally, the lipase activity of the intestine in the Gly group was significantly higher than that of the control group and the MA + Gly group, while the hepatopancreatic lipase decreased significantly. The amylase activity in the intestine and hepatopancreas of the Gly group was significantly lower than that of the control group. The trypsin activity in the hepatopancreas of the MA + Gly group was significantly higher than that of the Gly group. The Shannon diversity index in MA + Gly group was significantly lower than that in control group. At the phylum level, the abundance of the Campilobacterota in the MA + Gly group decreased. At the genus level, the proportion of the *Citrobacter and Flavobacterium* in the MA + Gly group decreased.

**Conclusion:** Gly has certain effects on the digestive tissue function, intestinal microbial diversity index and intestinal microbiota structure of *E. sinensis*, and MA can ameliorate the negative effects of Gly on *E. sinensis*.

**Summary**

• Glyphosate (Gly) changed the structure and function of digestive organs of *E. sinensis*.
• Gly changed the intestinal microbiota diversity and community structure.
• MA improved the structure and function of digestive organs caused by Gly.
• MA improved the intestinal microbiota diversity and community structure.

## 1. Introduction

The Chinese mitten crab (*Eriocheir sinensis*), also known as the river crab, is an economic freshwater crab species that is widely cultivated in China. In 2020, the annual output of *E. sinensis* reached approximately 775,887 tons [1]. Glyphosate (Gly) is the isopropyl amine salt of N-phosphonomethylglycine. Gly is utilized in aquaculture to eliminate weeds surrounding ponds. It may be can enter water bodies through irrigation, rainwater, and runoff, leading to adverse effects on aquatic animals [2–6]. However, there is a paucity of research on the effects of these products on *E. sinensis*.

The growth and development of *E. sinensis* are closely linked to their digestive function [7]. Numerous studies have shown that digestive enzymes are a crucial factor in evaluating animal digestive physiology [8]. Furthermore, the intestine serves as the primary organ for digesting food and absorbing nutrients, while the hepatopancreas plays a crucial role in detoxifying crustaceans [9]. Its tissue structure can also serve as an indicator of environmental impact to some extent [10].

Previous studies have shown that the intestinal microbiome has a significant impact on health and can contribute to various neurological diseases, including depression and anxiety and autism spectrum disorder (ASD) [11, 12]. Intestinal bacteria play a crucial role in synthesis vitamins and amino acids, as well as detoxifying exogenous toxins [13]. Studies have shown that exposure to pesticides can interfere with the host's intestinal microorganisms, causing translocation of these microorganisms and subsequent intestinal tissue damage [14].

Microbe-derived antioxidants (MAs) are a type of microbial preparation extracted from various beneficial bacteria through fermentation and concentration. This product is created through compound fermentation of beneficial microorganisms, such as *Bacillus subtilis*, *Clostridium butyricum*, and *Lactobacillus*. The process includes extraction, concentration, inactivation, freeze-drying, and other procedures [15]. MA contains various trace elements, including vitamin C, vitamin E, glutathione, and taurine. Current research shows that they can improve the antioxidant capacity of animals, enhance their immune function, have a good damage repair effect, and improve their reproductive ability [16, 17]. However, there is little research on *E. sinensis*.

The aims of this study is to alleviate the negative impact of Gly on aquaculture through nutritional feed regulation technology, reduce mortality and increase yield, and improve economic and social benefits.

## 2. Materials and Methods

### 2.1. Animals

All animals were handled according to the permits of the Animal Experiments Ethics Committee of Shanghai Ocean University for the care and use of laboratory animals (SHOU-DW-2021-088). The experiment crabs *E. sinensis* (100–110 g) were obtained from Chongming Island base of Shanghai Ocean University. The crabs were acclimated to laboratory conditions in a circulating water system, with the temperature kept between 20 and 23°C, pH between 7.6 and 7.8, and dissolved oxygen (DO) concentration at 6.3 ± 0.4 mg/L. They were fed once a day and kept on a 12-h day/night cycle for 1 week [18].

### 2.2. Experiment Designs and Sampling

For the experiment, 80 healthy and energetic crabs were randomly selected and cultured in blue boxes measuring 56 cm × 42 cm × 31 cm. PVC plastic pipes were provided as shelter. The experiment was divided into four groups: (1) The control group (control): the crabs were fed the commercial feed; (2) Gly treatment group (Gly): the crabs were lived in the Gly solution (Monsanto, USA) and the concentration of Gly at the 50% 96 h LC_50_ value (48.945 mg/L) [19]; (3) MA treatment group (MA): the crabs were fed the commercial feed supplemented with MA; and (4) Gly and MA treatment group (MA + Gly): the crabs were lived in the Gly solution and fed with the commercial feed supplement with MA. The crabs in each group were divided into three parallel groups. Half of the water was changed daily at 9:00, and Gly was added to ensure consistent concentration throughout the 7-day experiment. Before the end of the experiment, after 24 h of starvation, three crabs were randomly selected from each group for histological observation. The midgut, hindgut, and hepatopancreas were then taken and quickly stored in Bouin's solution for 24 h. Six crabs were randomly selected from each group and the midgut, hindgut, and hepatopancreas were taken and quickly stored in −20°C for digestive enzymes determination. Six crabs from the control group, MA group, and MA + Gly group were anesthetized on ice, and then the contents of their hindguts were washed out with saline and stored at −80°C for high-throughput sequencing.  Table 1 shows the formula and chemical composition information of the commercial feed.

### 2.3. Histological Analysis

After fixing the intestine and hepatopancreas with Bouin's solution for 24 h, we followed these steps for the embedding experiment: The tissue was dehydrated using a series of grades ethanol solutions with increasing concentrations: 75% for 30 min, 80% for 30 min, 90% for 30 min, and 95% for 30 min (repeated once). Finally, the tissue was dehydrated in 100% ethanol for 30 min (repeated once) before being transferred to a preprepared mixture of 1/2 ethanol and 1/2 xylene for 30 min. The tissue was immersed in xylene for 15 min (note that different tissues may require different transparency times). It was then transferred to a mixture of 1/2 xylene and 1/2 paraffin at 60°C for 30 min. The wax was permeated in paraffin liquid at 60°C for 2 h, and this process was repeated. Finally, the fully waxed tissue was placed into a prepared embedding box, embedded with paraffin liquid, and sliced 24 h later with a thickness of 5–7 μm. The resulting slice was stored in a 37°C incubator for future use. Remove the prepared sections for H.E. staining. Follow these specific steps: Immerse the slices in xylene for 10 min, repeating once. Then, immerse them in a solution consisting of half xylene and half 100% ethanol for 10 min. Subsequently, immerse them in 100% ethanol, followed by 95%, 80%, and 70% ethanol, and finally sterilized distilled water for 2 min, repeating once. For staining, place the slices in hematoxylin solution for 30 min, and then wash them slowly with running water for 20 min. Differentiate the slices by placing them in a 1% hydrochloric acid aqueous solution for several seconds until the hydrochloric acid solution turns red, then rinse with 0.1% sodium bicarbonate solution for 10 min. The tissue slices were treated with 5% ammonia for 30 s, followed by immersion in sterilized distilled water for 1 min. Subsequently, the slices were immersed in 70%, 80%, and 95% ethanol for 2 min each. After that, the slices were treated with Eosin dye solution for 30 s, followed by immersion in 95% and 100% ethanol for 2 min each. Finally, the slices were placed in a mixture of 1/2 xylene and 1/2 absolute ethanol for 5 min, followed by transfer to pure xylene for 5 min, repeated once. The sections were then sealed with neutral balsam. The sections were photographed using an Eclipse 80i microscope and photographic system (Nikon, Japan) [20].

The tissue area was calculated by ImageJ software. The specific operation is as follows: Open ImageJ software and set the correct scale bar. Select Analyze in the menu bar, then select Set Measurements, and then check the Area and Display label options. Open the image to be measured. Select the polygon tool in the toolbar and select the irregular area to be measured. Select the Analyze and Measure command, ImageJ will calculate and display the area of the selected area.

### 2.4. Determination of Digestive Enzymes

To determine the digestive enzymes, the thawed intestinal and hepatopancreatic tissues were mixed with crab normal saline in a 1:9 ratio. The mixture was homogenized on ice for 1 min and then centrifuged at 4°C and 12,000 r/min for 10 min. The supernatant was collected and stored in a centrifuge tube for further analysis. The same method was used for the hepatopancreas. After homogenization in the homogenizer, centrifugation was carried out. The middle layer was transferred to a new centrifuge tube and centrifuged for 10 min. The clear liquid from the middle layer was collected for measurement. Lipase, amylase, and trypsin were determined following the instructions provided by Nanjing Jiancheng Bioengineering Institute [6].

### 2.5. Intestinal Microbiota Diversity

The intestinal content samples stored at −80°C were sent to Shanghai Meiji Biomedical Technology Co., Ltd. for high-throughput sequencing analysis. Each purified PCR product was then constructed into a library using the Illumina MiSeq platform, and the resulting fragments were sequenced on the MiSeq PE300 platform. The sequencing data was graphically rendered using the I-Sanger cloud platform provided by Shanghai Meiji Biology Co., Ltd. [21].

### 2.6. Statistical Analysis

The mean ± S.D. was used to present all data. One-way analysis of variance (ANOVA) was performed using SPSS (V22.0, IBM Corporation, NY, USA) on the data marked with letters in some figures, followed by Tukey B's comparison tests. Statistical significance was considered at *p*  < 0.05. Different lowercase letters in the figure indicate significant differences among groups (*p*  < 0.05) [22].

## 3. Results

### 3.1. Histological Observation

#### 3.1.1. Histological Structure of Midgut and Hindgut of *E. sinensis*

**Figure 1:**
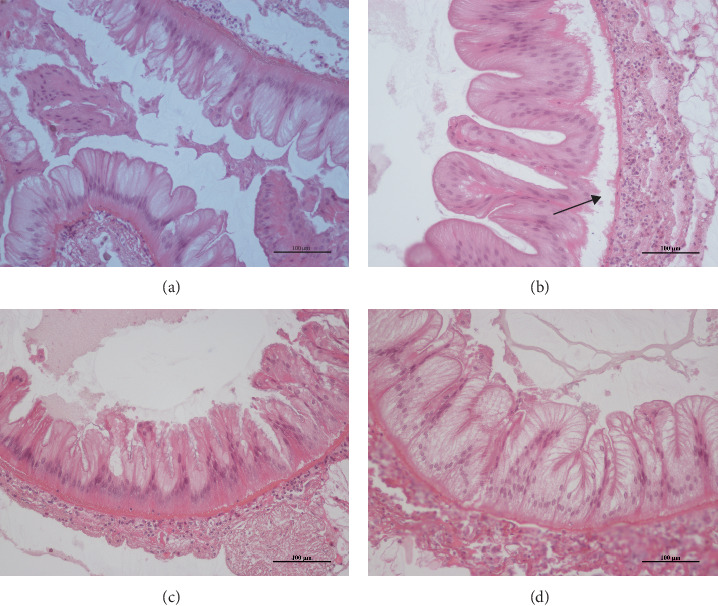
H.E. staining of midgut tissue of *Eriocheir sinensis*. Scale bar: 100 μm. Control group (A), Gly exposure group (B), MA feed group (C), and feeding MA under Gly exposure group (D). Gly, glyphosate; MA, microbe-derived antioxidant.

The H.E. Staining of the midgut showed that the mucosal folds of the midgut in the control group did not break away from the basement membrane, and a large number of mucosal folds fell off from the basement membrane in the Gly group, as shown by the black arrow in Figure 1B. In the MA + Gly group, the folds of the midgut mucosa of the Chinese mitten crab were not separated from the basement membrane (Figure 1).

**Figure 2:**
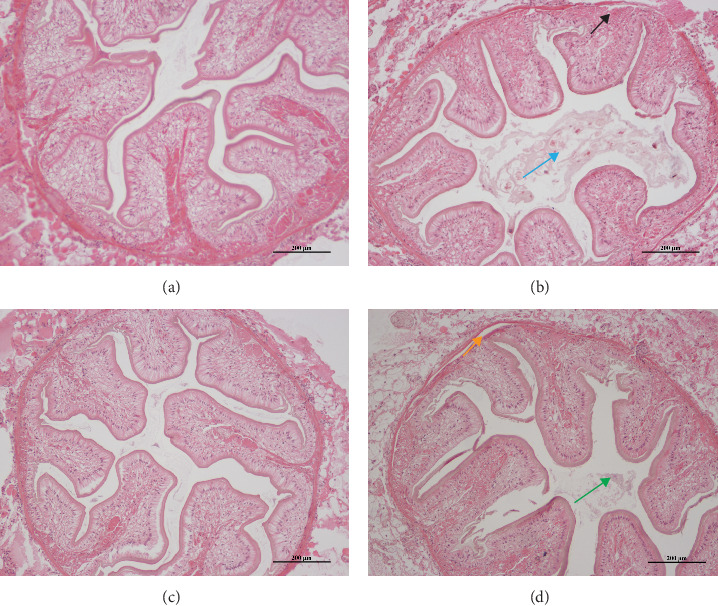
H.E. staining of hindgut tissue of *Eriocheir sinensis*. Scale bar: 200 μm. Control group (A), Gly exposure group (B), MA feed group (C), and feeding MA under Gly exposure group (D). Gly, glyphosate; MA, microbe-derived antioxidant.

The H.E. staining of hindgut results showed that compared with the control group, folds of intestinal mucosa fell off from the basement membrane in the Gly group (black arrow in Figure 2), and the proportion of fold area of hindgut mucosa decreased (blue arrow in Figure 2). In the MA + Gly group, the folds of intestinal mucosa also fell off (orange arrow in Figure 2), but the proportion of fold area of intestinal mucosa increased (green arrow in Figure 2) compared to the Gly group.

The cross-sectional area and mucosal fold area of the hindgut of *E. sinensis* were measured by ImageJ software.  Table 2 shows that the mucosal fold ratio of *E. sinensis* decreased significantly after Gly exposure (*p*  < 0.05) compared to the control group. Additionally, Table 2 indicates a significant increase in the mucosal fold ratio after feeding MA (*p*  < 0.05).

#### 3.1.2. Histological Structure of Hepatopancreas of *E. sinensis*

**Figure 3:**
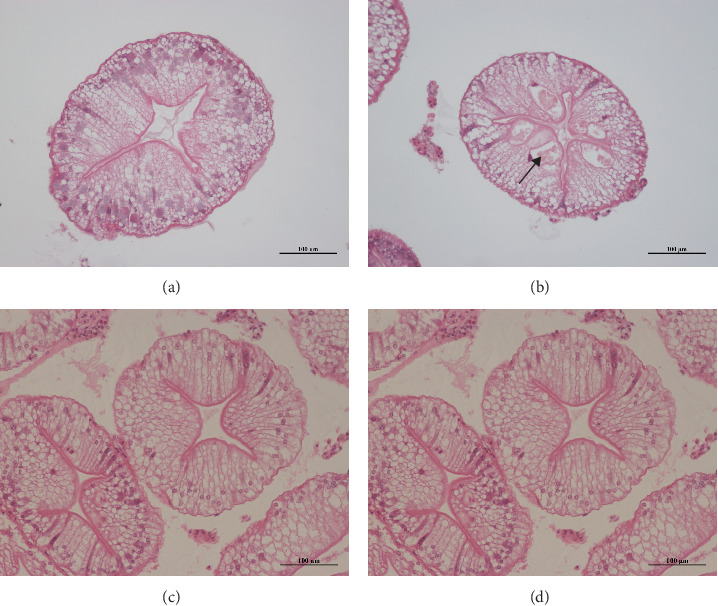
H.E. staining of hepatopancreas of *Eriocheir sinensis*. Scale bar: 100 μm. Control group (A), Gly exposure group (B), MA feed group (C), and feeding MA under Gly exposure group (D). Gly, glyphosate; MA, microbe-derived antioxidant.

The experiment's H.E. staining results showed that, compared to the control group, the Gly group had an increased number of B cells in the hepatopancreas and hepatic tubules, as well as a decreased lumen area (black arrow in Figure 3).

### 3.2. Digestive Enzymes

#### 3.2.1. Intestinal Digestive Enzymes Activity

**Figure 4:**
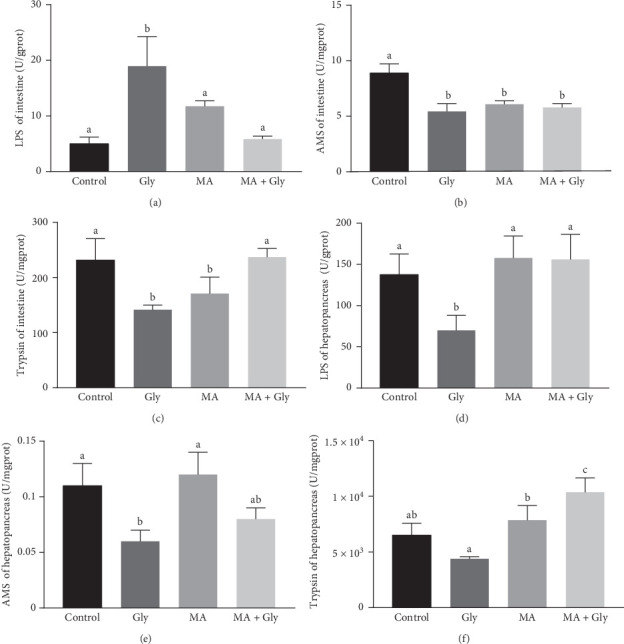
Effects of microbial antioxidants on digestive enzyme activities in intestine and hepatopancreas of *Eriocheir sinensis* exposed to Gly. Lipase (A), amylase (B), and trypsin (C) activities of intestine; lipase (D), amylase (E), and trypsin (F) activities of hepatopancreas. Gly, glyphosate; MA, microbe-derived antioxidant.

The lipase activity in the Gly group was significantly higher than that in the control group and the MA + Gly group (*p*  < 0.05). In contrast, amylase activity was significantly decreased in the other three groups compared to the control group (*p*  < 0.05). The trypsin activity in the Gly group was significantly lower than that in the control group (*p*  < 0.05). In contrast, the trypsin activity in the MA + Gly group was significantly higher than that in the Gly group (*p*  < 0.05). Additionally, the trypsin activity in the MA group was significantly decreased compared to the control group (*p*  < 0.05; Figure 4A–C).

#### 3.2.2. Hepatopancreas Digestive Enzymes Activity

The lipase activity of the hepatopancreas was significantly decreased in the Gly group compared to the control group (*p*  < 0.05). Additionally, the amylase activity of the hepatopancreas was significantly lower in the Gly group than in the control group (*p*  < 0.05). In contrast, the MA + Gly group and MA group had significantly higher lipase activity than the Gly group (*p*  < 0.05). Finally, the trypsin activity of the hepatopancreas was significantly higher in the MA + Gly group and MA group than in the Gly group (*p*  < 0.05; Figure 4D–F).

### 3.3. Intestinal Microbiota Diversity

#### 3.3.1. Analysis of Intestinal Microbiota Abundance and Diversity

After quality filtration using the Illumina MiSeq PE300 sequencing system, 800,314 effective sequences were generated from the intestinal microbiota. The total base number was 338,978,684 and the average sequence length was 424.1 bp. The control group had an average sequence number of 41,750 ± 7718, the MA group had an average sequence number of 43,291 ± 3010, and the MA + Gly group had an average sequence number of 48,314 ± 7986. The average sequence length was 424.86 ± 1.41, 424.64 ± 1.97, and 422.80 ± 6.98 for the control group, MA group, and MA + Gly group, respectively. The shortest sequence length and the longest sequence length were 217, 200, and 201 bp, and 515, 526, and 478 bp, respectively (Table 3).

**Figure 5:**
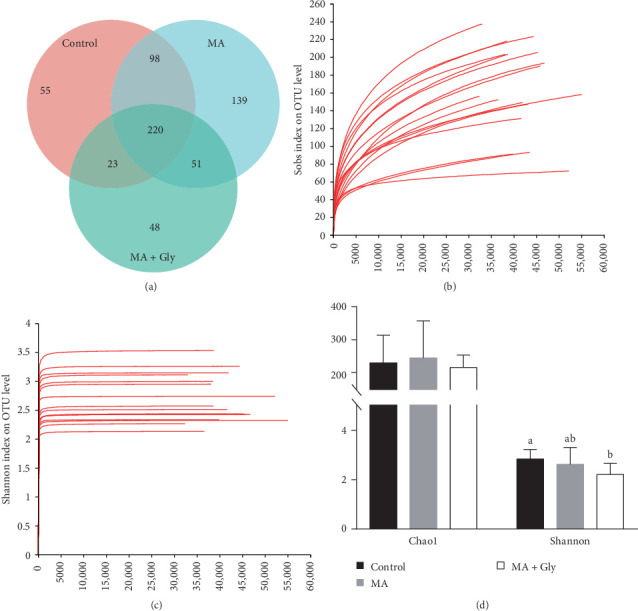
Effects of microbial antioxidants on the distribution of OTUs and alpha diversity of intestinal microbiota in *Eriocheir sinensis* exposed to Gly. (A) The distribution of OTUs; (B) rarefaction curves; (C) Shannon curves; (D) Chao1 richness and Shannon diversity index. Gly, glyphosate; MA, microbe-derived antioxidant.

Using the Silva database for bacteria and archaea, we compared representative sequences of OTUs and found a total of 1245. Following MA feeding, 220 OTUs were shared by all three groups (Figure 5A).

The dilution curve shows that as sequencing depth increased, the number of observed species and the Shannon curve gradually flattened, further verifying the subsequent analysis. Compared to the control group, the MA + Gly group showed a significant decrease in the Shannon diversity index (Figures 5b–d).

**Figure 6:**
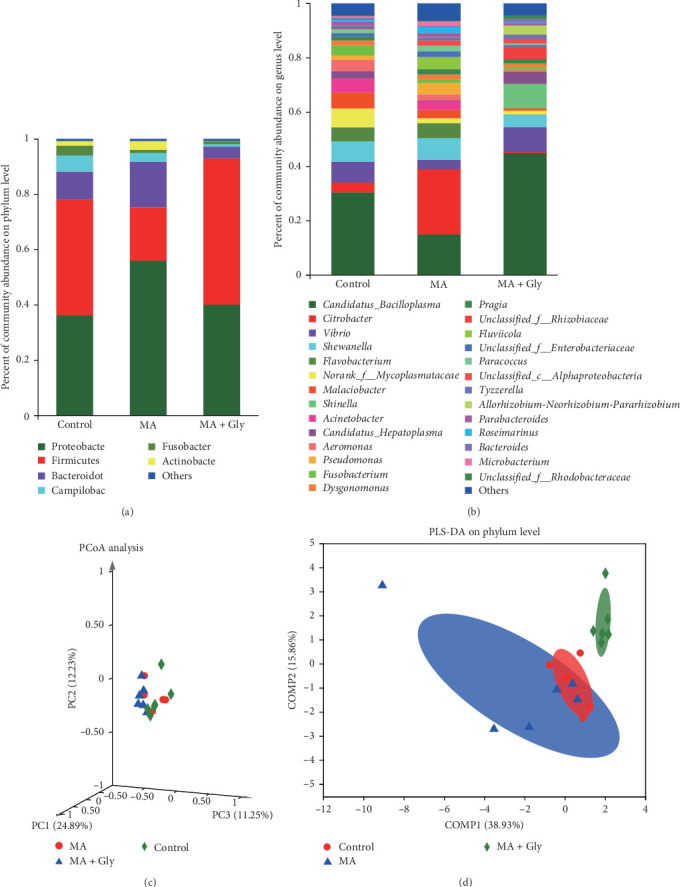
Effects of microbial antioxidants on the percentage of intestinal microbiota at phylum level (A) and genus level (B), and PCoA analysis (C) and PLS-DA (D) discriminant analysis of intestinal microbiota in *Eriocheir sinensis* exposed to Gly. Gly, glyphosate; MA, microbe-derived antioxidant.

The microbial samples were divided into three groups and sequenced. The microbial community structure consisted of 26 phyla, 52 classes, 123 orders, 208 families, 352 genera, and 489 species. The top 6 microbial communities at the phylum level were Proteobacteria, Firmicutes, Bacteroidota, Campilobacterota, Fusobacteriota, and Actinobacteria. Data analysis revealed a decrease in the proportion of Campylobacter community abundance in the MA + Gly group (Figure 6A). At the genus level, *Bacillus*, *Citrobacter*, *Shewanella*, *Flavobacterium*, and *Vibrio* were found to have high abundance. The abundance percentages of microbial communities differed significantly between *Citrobacter* and *Flavobacterium* among the three groups. In comparison to the control and MA groups, the abundance percentage of the *Citrobacter* and *Flavobacterium* communities in the MA + Gly group decreased (Figure 6B).

Figure 6C shows that the microbial sample community composition is highly similar among the control group, MA group, and MA + Gly group, as their spatial distance is relatively close. In Figure 6D, the three ellipses represent the three different groups, with the control group partially overlapping with the MA group, and the MA + Gly group being close to the other two groups.

#### 3.3.2. Prediction of Microbiota Metabolic Function

**Figure 7:**
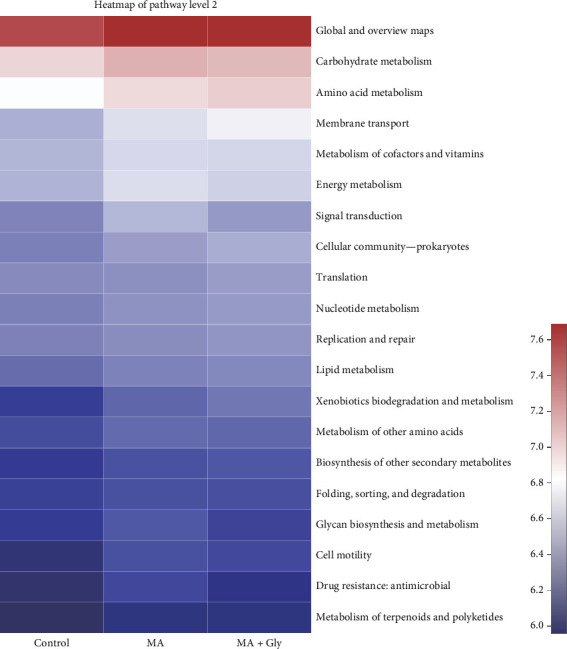
KEGG heatmap of metabolism function of *Eriocheir sinensis* predicted by PICRUSt. Gly, glyphosate; MA, microbe-derived antioxidant.

According to compared the prediction of microbial metabolic function of intestinal contents, it shows that the metabolic function of the top 20 of the relative abundance of intestinal microorganisms of *E. sinensis* is relatively close after feeding microbial preparations among the three groups (Figure 7). The intestinal flora of *E. sinensis* is relatively close and abundant in the three metabolites of energy metabolism, carbohydrate metabolism, and amino acid metabolism.

## 4. Discussion

Gly can cause the intestinal mucosa of the Chinese mitten crab to shed and reduce the area of the intestinal folds. The probiotics in MA feed can effectively counteract the damage caused by Gly. Yu [23] found that pymetrozine significantly affected the gastrointestinal digestive tissue lesions of crayfish. The abscission phenomenon of the mucosal fold may lead to the destruction of the peritrophic membrane and injury to the hindgut tissues, which may affect the digestive and absorptive capacity of *E. sinensis*. Albañil Sánchez et al. [24] found that Gly can cause severe histological damage in neotropical fish. After feeding MA, this study found that the middle intestinal mucosal fold did not desquamate from the intestinal basement membrane of *E. sinensis*, and the hindgut still desquamated slightly, but the area of the intestinal mucosal fold was significantly increased. This may be because the beneficial microorganisms in the diet play a regulatory role and do not have an obvious protective effect on the hindgut, the possible reason being that the experimental cycle is short. Zhou's study [25] shows that *B. subtilis* is beneficial to protect and increase the contact area between the intestinal mucosa and food and enhance the absorption and utilization efficiency of nutrients. Li et al. [26] showed that probiotics such as *B. subtilis* can improve and maintain the morphological structure and integrity of the intestine. A study in common carp found that feeding microbial diets could improve the height of the villus, increase the wrinkling of the foregut mucosa, and increase the intestinal absorption area [27].

MA feed can effectively resist hepatopancreas damage caused by Gly. The hepatopancreas of *E. sinensis* and other Decapoda crustaceans is an important organ for digestion and detoxification. When affected by foreign substances, their hepatopancreas suffers structural damage commensurate with the magnitude of the impact, which may to some extent reflect changes in the adaptability of organisms to the external environment [28]. This study found that the number of B cell transport vesicles in the hepatic tubules of *E. sinensis* increased after Gly exposure, indicating that Gly may have a toxic effect on *E. sinensis*. Hong et al. [29] proposed that deltamethrin and ammonia nitrogen metabolites may accumulate in B-cell transport vesicles and achieved partial detoxification by B-cell transport in vitro. After feeding MA, the number of B cells in the hepatic tubules of *E. sinensis* decreased significantly compared with the Gly exposure group, which may be because the MA feed contains a large number of probiotics, which can effectively improve the immune activity of carp and promote the growth and development of immune organs [27]. From a dietary point of view, the benefits of probiotics can not be replaced by any additives.

MA can alleviate the digestive damage caused by Gly and enable normal fat metabolism in the intestines of Chinese mitten crab. The activity of digestive enzymes directly reflects the ability of animals to digest and absorb nutrients and determines their growth and development rate [7]. Many studies have shown that digestive enzymes are affected by a variety of external factors such as ammonia nitrogen stress, salinity stimulation, organic pollutants, and so on [30–32]. In this study, after 7 days of Gly exposure, intestinal lipase of *E. sinensis* significantly increased and hepatopancreatic lipase significantly decreased. After Gly exposure, intestinal amylase, trypsin, and hepatopancreatic amylase of *E. sinensis* decreased significantly, indicating that Gly reduced the digestion and absorption capacity of starch and protein or Gly inhibited the feeding of *E. sinensis* and accelerated its carbohydrate and protein metabolism. Samanta et al. [33] showed that changes in digestive enzyme activity may be due to disruption of normal protein, carbohydrate, and lipid metabolism, particularly damage to digestive glands such as the stomach. By simulating human gastric and intestinal juices, high concentrations of Gly can interact with pepsin and trypsin, which can affect the digestive function of animals [34].

This study found that intestinal trypsin activity, hepatopancreatic lipase activity and trypsin activity of *E. sinensis* were significantly increased after feeding MA. The reason may be that a variety of probiotics in MA are involved in regulating the body's detoxification function. Studies have shown that lactic acid bacteria can adhere to gastrointestinal epithelial cells and mucus and enhance the digestive capacity of animal digestive organs. *B. subtilis* can maintain and adjust the balance of intestinal flora, enhance the immunity of animals, secrete a variety of digestive enzymes, and improve feed digestion and utilization [35, 36]. Zhang et al.'s [37] study showed that the addition of *B. subtilis* to water can significantly improve the activities of amylase and lipase in the intestine of juvenile sea cucumber. *Bacillus subtilis* in feed can stimulate the nonspecific immune response in aquatic animals, improve the morphology, and structure of the small intestinal mucosa and enable the intestinal tract to carry out normal fat metabolism [38].

This study found that after feeding MA, the total number of OTUs in the three groups of *E. sinensis* was higher, indicating that the microbial communities of the three groups had a high similarity. MA significantly improved the Chao1 richness of *E. sinensis* exposed to Gly. Previous studies showed that Gly significantly reduced the Chao1 richness of *E. sinensis* gut microorganisms [6], indicating that MA can reverse the imbalance of the bacterial community caused by Gly. Lee et al. [35] noted that the addition of *B. subtilis* to the feed can maintain a stable gut microbial community and promote animal growth and development. The probiotics added to the feed are colonized in the intestinal tract through the Biolog-ECO method, so as to change the abundance and structure of the original flora in the intestinal tract, promote the microbial communities in the intestinal tract of shrimp to maintain a stable state [39].

MA can reduce the relative abundance of pathogenic bacteria. After feeding MA, it was found that the proportion of Campylobacter in the community abundance decreased, and *Citrobacter* and *Flavobacterium* decreased significantly. Gao et al. [40] and Law et al. [41] pointed out that the common types of foodborne pathogens mainly include Listeria and Campylobacter, indicating that the reduction of Campylobacter is conducive to the healthy growth and development of *E. sinensis*. Studies have shown that citric acid bacteria are conditional pathogens, among which *Citrobacter fleundii* is a typical conditional zoonotic pathogen that can cause a range of diseases such as diarrhea, food poisoning, and secondary infection under the appropriate conditions [42, 43], while *Flavobacterium columnaris* in *Flavobacterium* is a pathogenic bacterium of aquatic animals that can cause grass carp, mandarin fish, carp, channel catfish, and other economic fish to be infected with gill rot disease [44]. It shows that MA can reduce the abundance of *pathogenic bacteria* such as campylobacter, citric acid bacilli, and yellow bacilli, so as to prevent *E. sinensis* from infection and improve economic benefits.

MA improves the intestinal microbial community structure of *E. sinensis* exposed to Gly. This study found that the spatial distribution of community OTU among the three groups of samples were relatively close, indicating that MA played a positive role in alleviating the differences in the distribution of intestinal microbial OTU caused by Gly, that is, MA improved the beta diversity of *E. sinensis*. For the comparative analysis of microbiota structure, according to the PLS-DA discrimination model, it is found that the distance distribution among the three groups of samples is close, indicating that MA improves the intestinal microbial community structure of *E. sinensis* exposed to Gly. Wang et al. [45] analyzed the intestinal microbial composition in the feces of infants with diarrhea and found that Lactobacillus can improve the intestinal microbial composition.

In this study, PICRUST was used to predict the metabolic function of the intestinal flora of *E. sinensis*. The results showed that carbohydrate metabolism, amino acid metabolism, and energy metabolism of the three groups of intestinal microbiota were more active after feeding MA, which was similar to our previous research results on *E. sinensis* [6]. In addition, previous studies showed that Gly exposure significantly affected the intestinal exogenous biodegradation and metabolic function, carbohydrate metabolic function, and amino acid metabolic function of *E. sinensis* [6]. After feeding MA, all metabolic functions of *E. sinensis* returned to normal levels.

In this paper, we investigated the structural damage of Gly to the digestive tissues of *E. sinensis* by histology, used high-throughput sequencing technology to investigate for the first time the effect of Gly herbicide on the intestinal microbiota, and reversed the damage caused by Gly to the *E. sinensis* by feeding supplemental feed. It was found that feeding microbe derived antioxidants could improve the stress resistance of *E. sinensis*. Thereby, increasing the yield and economic benefits of and provide a reference for the culture of *E. sinensis*. Finally, the author suggested that Gly should be avoided in the culture of *E. sinensis*.  Table 4 lists all the abbreviations used in the manuscript and their full names.

## 5. Conclusion

Gly induced intestinal and hepatopancreatic tissue damage in *E. sinensis* and affected the digestive system by impairing intestinal and hepatopancreatic function. During Gly stress, the digestive system of *E. sinensis* was involved in the response. Gly can significantly reduce the intestinal microbial Chao1 richness index and Shannon diversity index, alter the structure of the intestinal microbial community and negatively affect the metabolic function of the flora. MA can improve the digestion and absorption capacity and immune capacity and significantly improve the intestinal microbial diversity, community structure, and flora metabolic function of *E. sinensis*, so as to improve disease resistance, reduce unnecessary losses, and improve economic benefits. Overall, MA can effectively alleviate the digestive damage and gut microbiota imbalance caused by Gly in Chinese mitten crab.

## Figures and Tables

**Table 1:** Ingredients and proximate composition of the commercial feed (% dry matter).

Ingredient	Content
Soybean meal	15.50
Peanut meal	8.00
Rapeseed meal	18.00
Cotton meal	7.00
Fish meal	7.00
Wheat flour	28.30
Yeast meal	2.00
Squid powder	2.00
Phosphatide oil	2.00
Fish oil	1.50
Pork lard	1.50
Mineral mix	0.30
Vitamin mix	1.20
Ca(H_2_PO_4_)_2_	1.00
Choline chloride	0.40
Dishulin	0.10
Bentonite	4.00
Salt	0.20
Total	100.00
Moisture	11.45
Crude protein	34.56
Crude lipid	8.34
Ash	9.15

**Table 2:** Cross sectional area and folds area of hindgut in *Eriocheir sinensis*.

Groups	Cross sectional area of intestine (μm^2^)	Fold area of intestinal mucosa (μm^2^)	F/C
Control	1,255,375 ± 50,003	1,077,550 ± 76,780	0.86 ± 0.03^a^
Gly	1,348,812 ± 268,626	967,449 ± 191,003	0.72 ± 0.001^b^
MA	1,082,880 ± 95,912	928,421 ± 85,418	0.86 ± 0.002^a^
MA + Gly	1,421,255 ± 268,178	1,148,548 ± 218,994	0.81 ± 0.001^c^

*Note*: Different letters in the table indicate that same index of this group is significantly different from that of other groups.

Abbreviations: Gly, glyphosate; MA, microbe-derived antioxidant.

**Table 3:** Sequencing information statistics of intestinal microorganisms in *Eriocheir sinensis* under different treatments.

Groups	Sequence number	Mean length (bp)	Min length (bp)	Max length (bp)
Control	41,750 ± 7718	424.86 ± 1.41	217	515
MA	43,291 ± 3010	424.64 ± 1.97	200	526
MA + Gly	48,314 ± 7986	422.80 ± 6.98	201	478

Abbreviations: Gly, glyphosate; MA, microbe-derived antioxidant.

**Table 4:** List of abbreviations in manuscripts.

Abbreviations	Full name
MAs	Microbe-derived antioxidants
Gly	Gly
MA + Gly	Gly and MA treatment
*E. sinensis*	*Eriocheir sinensis*
RD	Roundup

## Data Availability

The data are available with the corresponding author of this publication upon reasonable request.
